# Establishment of Protein Delivery Systems Targeting Podocytes

**DOI:** 10.1371/journal.pone.0011837

**Published:** 2010-07-29

**Authors:** Wen Chih Chiang, Tessa M. Geel, Mehmet M. Altintas, Sanja Sever, Marcel H. J. Ruiters, Jochen Reiser

**Affiliations:** 1 Department of Medicine, Massachusetts General Hospital, Boston, Massachusetts, United States of America; 2 Department of Internal Medicine, National Taiwan University Hospital, Taipei, Taiwan; 3 Department of Pathology and Medical Biology, Groningen University Institute for Drug Exploration (GUIDE), University Medical Center Groningen (UMCG), Groningen, The Netherlands; 4 Division of Nephrology and Hypertension, Leonard Miller School of Medicine, University of Miami, Miami, Florida, United States of America; 5 Synvolux Therapeutics BV, Groningen, The Netherlands; University of Pittsburgh Medical Center, United States of America

## Abstract

**Background:**

Podocytes are uniquely structured cells that are critical to the kidney filtration barrier. Their anatomic location on the outer side of the glomerular capillaries expose podocytes to large quantities of both plasma and urinary components and thus are reachable for drug delivery. Recent years have made clear that interference with podocyte-specific disease pathways can modulate glomerular function and influence severity and progression of glomerular disease.

**Methodology/Principal Findings:**

Here, we describe studies that show efficient transport of proteins into the mammalian cells mouse 3T3 fibroblasts and podocytes, utilizing an approach termed profection. We are using synthetic lipid structures that allow the safe packing of proteins or antibodies resulting in the subsequent delivery of protein into the cell. The uptake of lipid coated protein is facilitated by the intrinsic characteristic of cells such as podocytes to engulf particles that are physiologically retained in the extracellular matrix. Profection of the restriction enzyme *Mun*I in 3T3 mouse fibroblasts caused an increase in DNA degradation. Moreover, purified proteins such as *β*-galactosidase and the large GTPase dynamin could be profected into podocytes using two different profection reagents with the success rate of 95–100%. The delivered *β*-galactosidase enzyme was properly folded and able to cleave its substrate X-gal in podocytes. Diseased podocytes are also potential recipients of protein cargo as we also delivered fluorophore labeled IgG into puromycin treated podocytes. We are currently optimizing our protocol for *in vivo* profection.

**Conclusions:**

Protein transfer is developing as an exciting tool to study and target highly differentiated cells such as podocytes.

## Introduction

Podocyte disease often results in proteinuria, a disease process that can result from genetic mutations [1]–[5] or as the consequence of signaling pathways that are in disarray due to the deleterious action of cytosolic cathepsin L [6]. In both settings, it is desirable that podocytes can be reached by pharmacological interventions. While it is possible to reach podocytes through gene transfer [7] or small molecules [7], the delivery of functional peptides or proteins inside podocytes have not been established yet. In order to envision effective treatment for podocyte diseases, numerous drug delivery approaches need to be established and potentially combined. Moreover, protein delivery might be safer when compared to gene or chemical therapy. Having such a cargo system, it may allow the delivery of proteins or peptides into podocytes that inhibits cytosolic cathepsin L activity or protect cleavage targets from proteolysis. Podocytes are uniquely structured cells that are exposed to large quantities of both plasma and urinary components. These characteristics predispose podocytes to be amenable for protein delivery into intracellular space to interfere with podocyte-specific disease pathways, modulate glomerular function and alter the course of proteinuric renal diseases. Our laboratory has developed a protein delivery protocol that allows the uptake of exogenous protein that can exert its function in multiple cells including podocytes.

## Results

### DNA fragmentation after profection of NIH3T3 cells with restriction enzyme *Mun*I

**Figure 1:**
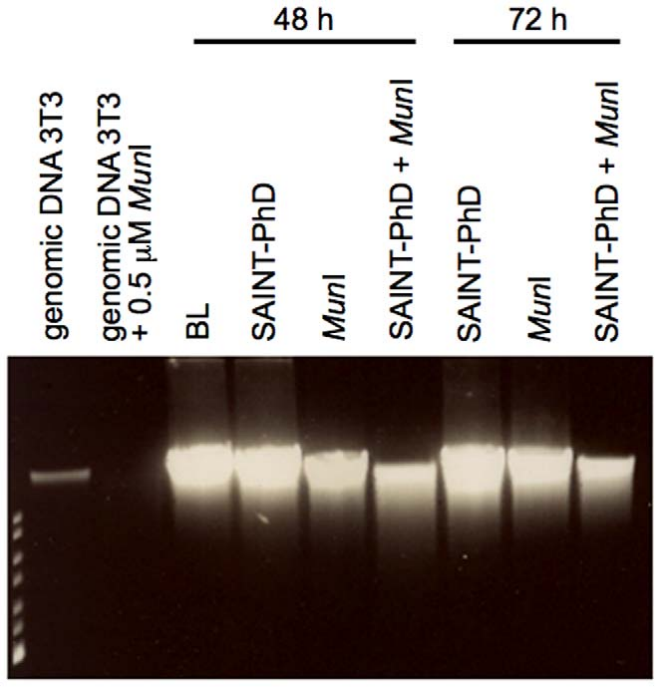
Restriction enzyme *Mun*I delivery into mouse 3T3 fibroblasts. NIH3T3 mouse fibroblasts were seeded in a 48 well plate (20,000 cells/400 µl per well) and treated with 0.5 µM *Mun*I (48 h) or 0.05 µM *Mun*I (72 h) alone or in complex with SAINT-PhD. Forty-eight and 72 h after incubation cells were collected and DNA fragmentation analysis was performed. BL: blank, untreated cells.

To analyze if it was feasible to transport functional proteins into cultured cells, we started out using mouse 3T3 fibroblasts that are known to be easily transfectable with plasmid DNA [8]. We tested the potential of transporting an apoptotic inducer such as the restriction endonuclease *Mun*I into the cells using SAINT-PhD as a carrier. Forty-eight and 72 hours after profection, cells treated with SAINT-PhD plus *Mun*I showed decreased levels of genomic DNA compared to control cells (untreated) or cell that received SAINT-PhD only without *Mun*I (Figure 1). These results indicate that the transferred restriction enzyme *Mun*I can exert its function and cleave genomic DNA but only in the presence of the lipid carrier SAINT-PhD.

### Profection of functional β-galactosidase in wild type mouse podocyte

**Figure 2:**
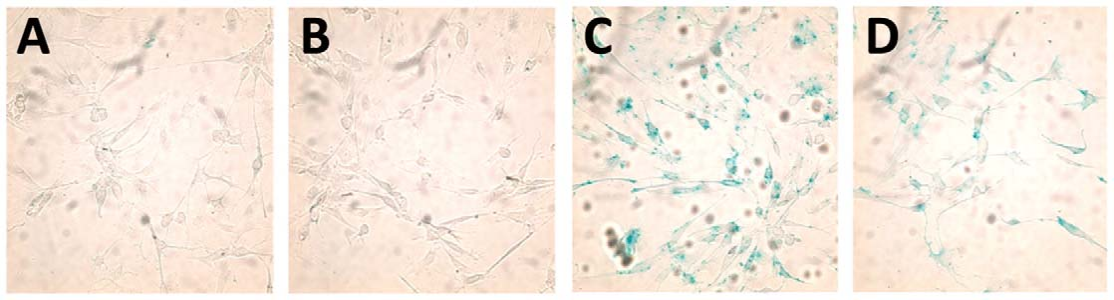
β-galactosidase was delivered into podocyte using SAINT-PhD and Chariot. Cells treated with only carrier agent (shown for SAINT-PhD, A) or β-galactosidase without any carrier agent (B) did not show blue staining. The delivered enzyme exerted its function as it cleaved X-gal substrate and turned the cells into blue color: Profection using SAINT-PhD (C) or Chariot (D) to carry β-galactosidase into podocytes.

In our next sets of experiments, we utilized 2 different profection reagents (SAINT-PhD and Chariot) as carriers to analyze transport of β-galactosidase into differentiated podocytes. Four hours after the application of the particles to the medium of podocytes, the cells were incubated with X-gal substrate to identify podocytes that have taken up enzymatically active β-galactosidase. Both carriers allowed the transport of active β-galactosidase into podocytes but with different efficiency. We found larger than 90% blue cells using SAINT-PhD and about 80% of blue podocytes using Chariot (Figure 2). The difference may lie in the different susceptibility of the carriers to serum. Intracellular delivery of proteins by SAINT-PhD is not significantly affected by the presence of serum as shown before [9]. However, in both cases, profection of active β-galactosidase into podocytes was achievable with high yield and was detectable even 4 days after profection.

### Delivery of immunoglobulin with SAINT-PhD in puromycin treated mouse podocyte

**Figure 3:**
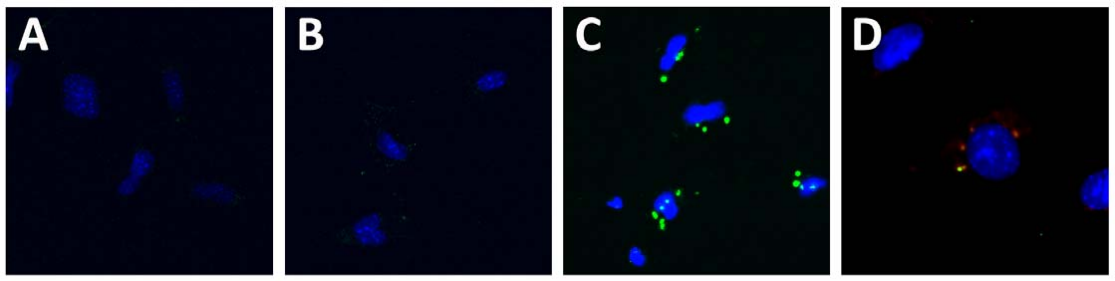
Transfer of immunoglobulin into PAN-pretreated mouse podocytes. Cells treated with only carrier agent (shown for SAINT-PhD, A) or IgG alone (B) did not have any uptake. In contrast, IgG packed into SAINT-PhD carriers allowed uptake of FITC-labeled IgG by podocytes. Cells treated with SAINT-PhD and IgG (C) showed punctuated staining in cytoplasm (green) that co-localized in part with lysosomes which were labeled with LAMP2 antibody (red) resulting a yellow overlap (D).

Since it is desirable to profect normal and diseased podocytes in order to extend options for novel therapeutics we evaluated the potential of antibody uptake in podocytes using SAINT-PhD and IgG. Interestingly, we were unable to deliver unpacked IgG or packed IgG into healthy podocytes but pretreatment of podocytes for 24 hour with the nephrotoxic stimulant puromycin aminonucleoside increased the uptake capacity for SAINT-PhD packed IgG. Most of the transferred IgG shortly after uptake was located in the membrane bound lysosomal compartment as seen by the co-labeling of IgG with the lysosomal membrane marker LAMP2 (Figure 3).

### Profection of dynamin into wild type podocyte

**Figure 4:**
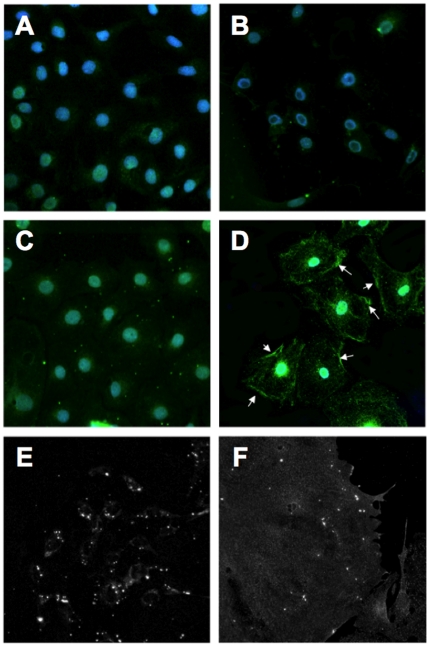
Delivery and sorting of dynamin GTPase into podocytes. Carrier alone (SAINT-PhD, A) or purified dynamin without carrier (B) was not taken up into podocytes. However, His-tagged dynamin packed into SAINT-PhD resulted in uptake and proper sorting of dynamin to the plasma membrane 12 hours after profection (C, low concentration; D, high concentration, arrows). Earlier time-points after profection (2 hours) start to show intracellular dynamin still captured in vesicles (E, low power view; F, high power view).

The large GTPase dynamin has recently been shown to be one of the main targets of cytoplasmic cathepsin L leading to the foot process effacement and proteinuria after being cleaved [6]. Stabilization of dynamin is thus one approach to rescue podocyte function as shown by gene delivery of cleavage resistant dynamin into mouse podocytes [6]. Thus, we tested profection of full length dynamin into cultured podocytes. Purified neuronal dynamin that carries a His-tag (His-dyn1) was packed into SAINT-PhD carrier and applied to the podocyte cell culture medium. His-tagged dynamin was detected with an anti-His antibody inside podocytes within 4 hours after profection (Figure 4). Of note, dynamin was correctly sorted to plasma membrane and profection efficiency was increasing with higher doses of purified dynamin packed in SAINT-PhD carriers.

## Discussion

Podocyte biology has made substantial progress over the past few years ranging from ground braking genetic studies to detailed molecular analyses regarding the mechanisms of proteinuria and podocyte injury [10]. Novel therapeutic approaches lie in the delivery of genes that encode for proteins that are resistant during podocyte injury [6], [11] or target disease pathways with the use of small molecules [7], [11]. An additional approach would be to deliver entire and functional proteins that can alter podocyte function under disease conditions. In this study, we have investigated a novel technique called profection. This technique uses lipid vesicles as carriers. It is a simple technique that works well for cultured podocytes. So far, the effects and efficiency vary for different types of cargo proteins and we need to investigate further how to optimize delivery and how to successfully use this approach *in vivo*. Several agents have been developed to deliver protein into cells, such as HIV-1 TAT [12], penetran 1 [13] and VP22 [14]. In addition, there is increasing use of nanogels carrying proteins into tissues [15], [16]. The above agents differ from SAINT-PhD and Chariot in that they need to be covalently bound to the macromolecules to be delivered [17]. The chemical reaction of covalent binding might denature the protein to be delivered and inactivate the molecules. In summary, we demonstrated that it is possible to deliver functional proteins into cultured podocytes and other cells using SAINT-PhD or Chariot. This approach bypasses the transcription-translation process of gene therapy. However, not all type of proteins can be transferred at equal amounts and efficiency. With different proteins, the reagents have different abilities to carry proteins into cells. Even the condition of cells (diseased versus healthy) is a factor influencing the uptake of the protein-carrier complex (e.g., IgG in this study). This study demonstrates the proof-of-concept for SAINT-PhD or Chariot to deliver functional proteins into podocytes and appears to be very promising for future applications including *in vivo* studies. Cargo packed in SAINT-PhD can result in sizes typically from 125–1700 nm depending on cargo (protein size). The filtration barrier in the kidney can classically restrict proteins or particles that are larger than albumin because the pore sizes of the podocyte slit membrane are roughly this size (4 nm by 14 nm). Based on this size range, packed cargo is not routinely allowed to pass the barrier. However, in glomerular kidney disease, there is a breakdown of the size and charge-selective barrier and cargo might pass the barrier relatively easily then. In addition, given the strong endocytosis and pinocytosis capability of podocytes [18], an uptake of cargo that is deposited in the glomerular basement membrane is probably the main path of podocyte entry. Our prediction would be that it is not required for cargo to be filtered. Though *in vitro* results are encouraging, it will require extensive research before effective *in vivo* therapies using podocyte protein delivery systems might come into use for treatment of podocyte-derived kidney diseases in humans. Among the need to test profection in the living animal, it will also become necessary to find out which factors influence uptake of cargo to optimize intracellular delivery and understand why not all cargo enter under the same conditions (e.g., dynamin versus IgG).

## Materials and Methods

Podocyte were cultured as previous described [6]. Differentiated wild type mouse podocytes (50,000 to 100,000 per well were cultured on coverglasses and subjected to PBS or 50 µg/mL of puromycin aminonucleoside (PAN) treatment for 24 hours. Different amount of fluorescence labeled immunoglobulin IgG (3–15 µg), His-tagged dynamin1 (1–3 µg), β-galactosidase (0.5–3 µg, Active Motif, Carlsbad, CA, USA) were delivered into podocyte using SAINT-PhD (Synvolux Therapeutics B.V., The Netherlands) or Chariot (Active Motif) and packed as per provided manufacturers' protocols. As negative controls, dynamin protein was also incubated without carrier at concentrations of 5–10 µg protein/6 well. Mouse 3T3 fibroblasts were cultured in were cultured in Dulbecco's modified Eagle's medium (DMEM) supplemented with 50 µg/ml gentamycine sulfate, 2 mM L-glutamine, 10% FBS.

For profection experiments, the cells were seeded in 48 well plates and profected with 0.5 µM or 0.05 µM *Mun*I the following day. Forty-eight and 72 h after profection, the cells were harvested and DNA fragmentation was carried out as described previously [19]. These protocols do not include covalent binding of cargo protein to the carrier reagent. For Chariot profection, the ratio of protein/reagent was 1–3/3.5 (µg/µL) in 0.5 mL of serum free culture medium in a 12 well plate. Protein and reagent were mixed and incubated at room temperature for 30 minutes. Then, the mixture was added into the culture medium. Four or 48 hours after profection, cells were fixed and labeled with suitable antibody, examined by microscopy or subjected to FACS analysis after labeling. Cells were examined with phase contrast or confocal microscopy. β-galactosidase activity staining was performed using the staining kit as protocol provided (Active Motif). DNA fragmentation was analyzed with staining with propidium iodide, followed by FACS examination.
